# Mapping the Escherichia coli DnaA-binding landscape reveals a preference for binding pairs of closely spaced DNA sites

**DOI:** 10.1099/mic.0.001474

**Published:** 2024-07-16

**Authors:** Anne M. Stringer, Devon M. Fitzgerald, Joseph T. Wade

**Affiliations:** 1 Wadsworth Center, New York State Department of Health, Albany, New York, USA; 2 Department of Biomedical Sciences, School of Public Health, University at Albany, SUNY, Albany, New York, USA; 3 RNA Institute, University at Albany, SUNY, Albany, New York, USA

**Keywords:** ChIP-seq, DnaA, DnaA box

## Abstract

DnaA is a widely conserved DNA-binding protein that is essential for the initiation of DNA replication in many bacterial species, including *Escherichia coli*. Cooperative binding of ATP-bound DnaA to multiple 9mer sites (‘DnaA boxes’) at the origin of replication results in local unwinding of the DNA and recruitment of the replication machinery. DnaA also functions as a transcription regulator by binding to DNA sites upstream of target genes. Previous studies have identified many sites of direct positive and negative regulation by *E. coli* DnaA. Here, we use a ChIP-seq to map the *E. coli* DnaA-binding landscape. Our data reveal a compact regulon for DnaA that coordinates the initiation of DNA replication with expression of genes associated with nucleotide synthesis, replication, DNA repair and RNA metabolism. We also show that DnaA binds preferentially to pairs of DnaA boxes spaced 2 or 3 bp apart. Mutation of either the upstream or downstream site in a pair disrupts DnaA binding, as does altering the spacing between sites. We conclude that binding of DnaA at almost all target sites requires a dimer of DnaA, with each subunit making critical contacts with a DnaA box.

## Data availability

ChIP-seq data are available at EBI ArrayExpress using accession number E-MTAB-12223.

## Introduction

DnaA is a highly conserved DNA-binding protein that is required for the initiation of DNA replication in many bacterial species [1,2]. DnaA exists in two nucleotide-bound states: DnaA–ATP and DnaA–ADP [2]. In *Escherichia coli*, both DnaA–ATP and DnaA–ADP bind multiple 9 bp high-affinity ‘DnaA box’ DNA sites (consensus: TTATCCACA) at the origin of replication (*oriC*) [3]. Binding of DnaA to DnaA boxes at *oriC* facilitates binding of DnaA–ATP to nearby low-affinity sites [4]. DnaA–ATP oligomerization across *oriC* results in local unwinding of the DNA and recruitment of the DnaG primase, DnaB helicase and DNA polymerase holoenzyme [2,5].

Following replication initiation, multiple independent mechanisms prevent immediate reinitiation. First, DnaA–ATP bound at *oriC* is converted into DnaA–ADP by a complex of Hda, the *β* clamp (DnaN) and DNA [6]. Second, SeqA binding to hemimethylated GATC sequences at *oriC* prevents reassociation of DnaA [7]. Third, SeqA binding to hemimethylated GATC sequences at the *dnaA* promoter represses transcription of *dnaA* [8]. Fourth, binding of DnaA to a strong site known as *datA*, upstream of *yjeV*, results in the titration of a substantial proportion of DnaA [9,10] and promotes conversion of *datA*-bound DnaA–ATP into DnaA–ADP [11]. DnaA–ADP is converted back to DnaA–ATP following interactions between DnaA monomers bound at two loci termed DnaA-reactivating sequences 1 and 2 (*DARS1* and *DARS2*), upstream of *uvrB* and *mutH*, respectively [12]. DnaA–ADP may also be converted into DnaA–ATP by interactions with acidic phospholipids [13].

In addition to its role in DNA replication initiation, DnaA regulates the transcription initiation of several genes by binding to sites in promoter regions [14,15]. *nrdA*, *glpD*, *polA* and *fliC* have been described as positively regulated DnaA targets [14,16,18]; *mioC*, *uvrB*, *aldA*, *guaB*, *proS*, *rpoH*, *iraD* and *dnaA* itself have been described as negatively regulated DnaA targets [14,19,26]. The mechanism of regulation by DnaA, especially that of transcription activation, is poorly understood. In addition to regulating transcription initiation, DnaA has been described to promote transcription termination by acting as a roadblock to elongating RNA polymerase when bound to DNA within a transcribed region [27,29]. Binding of DnaA to regulatory DNA sites likely changes as a function of cell cycle progression, as the level of DnaA–ATP and DnaA binding to *oriC* change [14,15].

## Results and Discussion

To facilitate Chromatin Immunoprecipitation coupled with DNA sequencing (ChIP-seq), we chromosomally tagged *dnaA* at its native locus with a C-terminal Sequential Peptide Affinity (SPA) tag [30]. We observed no significant difference in RNA levels for three DnaA-regulated genes (*nrdA*, *uvrB* and *polA*) between tagged and untagged strains (Fig. S1, available in the online version of this article; *t*-test, *p* = 0.21, 0.09 and 0.18 for *nrdA*, *uvrB* and *polA*, respectively), consistent with tagged DnaA retaining full regulatory function. We used ChIP-seq to map the association of SPA-tagged DnaA with the *E. coli* genome. As a control, we used ChIP-seq to map the association of SPA-tagged AcpS, a protein that retains function with the SPA-tag (*acpS* is an essential gene [31] and the tagged strain grows equivalently to wild-type *E. coli*) but is not expected to bind DNA [32]. By comparison of ChIP-seq data from the DnaA-SPA strain to those from the control AcpS-SPA strain (see Methods), we identified 16 DnaA-bound regions (Table 1). These regions include previously described DnaA-binding sites at *oriC* and upstream of *mioC*, *yjeV* (*datA*), *uvrB* (*DARS1*), *mutH* (*DARS2*), *nrdA*, *rpoH*, *dnaA* and *polA*. Putative, novel sites of DnaA association were observed upstream of *purH*, *nrdD* and *rne*, and within *dnaN*, substantially expanding the known DnaA regulon. We did not detect an association of DnaA with previously described sites upstream of *aldA*, *glpD*, *fliC*, *proS* and *iraD*, or within *guaB*, strongly suggesting that the associated genes are not part of the DnaA regulon.

**Table 1.** List of DnaA-bound regions identified by ChIP-seq

Genome **coordinate∗**	Associated gene/**l**ocus*	Coverage **relative** to **c**ontrol**†**	Double DnaA box**‡**
813 331	*DARS1*	16	c**T**A**AT**A**CACA**tgg**TTATCCACA**g
1 144772	(*yceQ*) *rne*	5	t**TTATC**AC**C**Cgc**TTA**CT**CACA**g
2344805	*nrdA*	14	g**TTATCCACA**aag**TTAT**G**CAC**Tt
2969124	*DARS2*	78	g**TT**C**T**T**CACA**actC**TATCCACA**g
2969306	*DARS2*	199	same as above
2969501	*DARS2*	213	tC**T**TA**C**AC**CA**tg**TTATCCACA**g
3600944	*rpoH*	4	t**TTATCCACA**ag**TT**CAATG**CA**a
3882238	(*dnaN*)	5	na
3883968	*dnaA*	5	t**TTATCCACA**ggaC**T**T**TCCA**G**A**a
3925806	*oriC*	218	c**TT**CCTG**ACA**gag**TTATCCACA**g
3925943	*oriC*	174	same as above
3926498	*mioC*	25	t**TTA**AT**C**C**CA**tac**TT**T**TCCACA**g
4046831	*polA*	123	t**TTAT**G**CACA**aag**TTATCCACA**t
4207740	*purH*	124	t**TTA**CG**CACA**gag**TTATCCACA**a
4392740	*datA*	213	c**TT**G**T**AA**ACA**gag**TTATCCACA**g
4462739	*nrdD*	62	t**TTA**AGA**ACA**gg**TTATCCACA**g

a *Genes in parentheses indicate an intragenic DnaA-bound region. Genes not in parentheses start close to an upstream DnaA-bound region.

b †ChIP-seq coverage for DnaA-SPA normalized to control data for AcpS-SPA.

c ‡Putative Ddouble DnaA boxes located within 200 bp of the indicated genome coordinate. Bases within a DnaA box are shown in upper-case. Bases matching the DnaA box consensus (TTATCCACA) are indicated byin bold, underlined text. No Ddouble DNA box was identified for the DnaA-bound region within *dnaN*. Two of the DnaA-bound regions at *DARS2* were associated with the same Ddouble DnaA box, as were the two regions at *oriC*.

* Genome coordinate of MG1655 (NCBI accession number: NC_000913.2)

Our data suggest that DnaA regulates the transcription of *purH*, *nrdD* and *nrdG*, which are genes involved in nucleotide biosynthesis. DnaA has been shown previously to regulate the transcription of *nrdA*, which encodes a component of the class Ia ribonucleotide reductase (RNR) [14,17]. DnaA-binding sites have been predicted upstream of *nrdD* [14,33] but not previously demonstrated experimentally. *nrdD* and its operonic gene *nrdG* encode subunits of the class III RNR [33]. Thus, components of two different RNR enzymes are likely regulated by DnaA.

The binding of DnaA upstream of *rne* has not been described previously. *rne* encodes RNase E, the major RNase in *E. coli*, that functions in a complex known as the degradosome [34]. No direct connection between RNase E and DNA replication has been described, although RNase E is required for specific processing of at least one mRNA encoding a DNA replication protein (DnaG) [35]. *dnaN* encodes the sliding clamp that facilitates efficient DNA replication. While the function of DnaN is closely tied to that of DnaA, we have not determined whether the DnaA site within *dnaN* is regulatory.

**Fig. 1.**
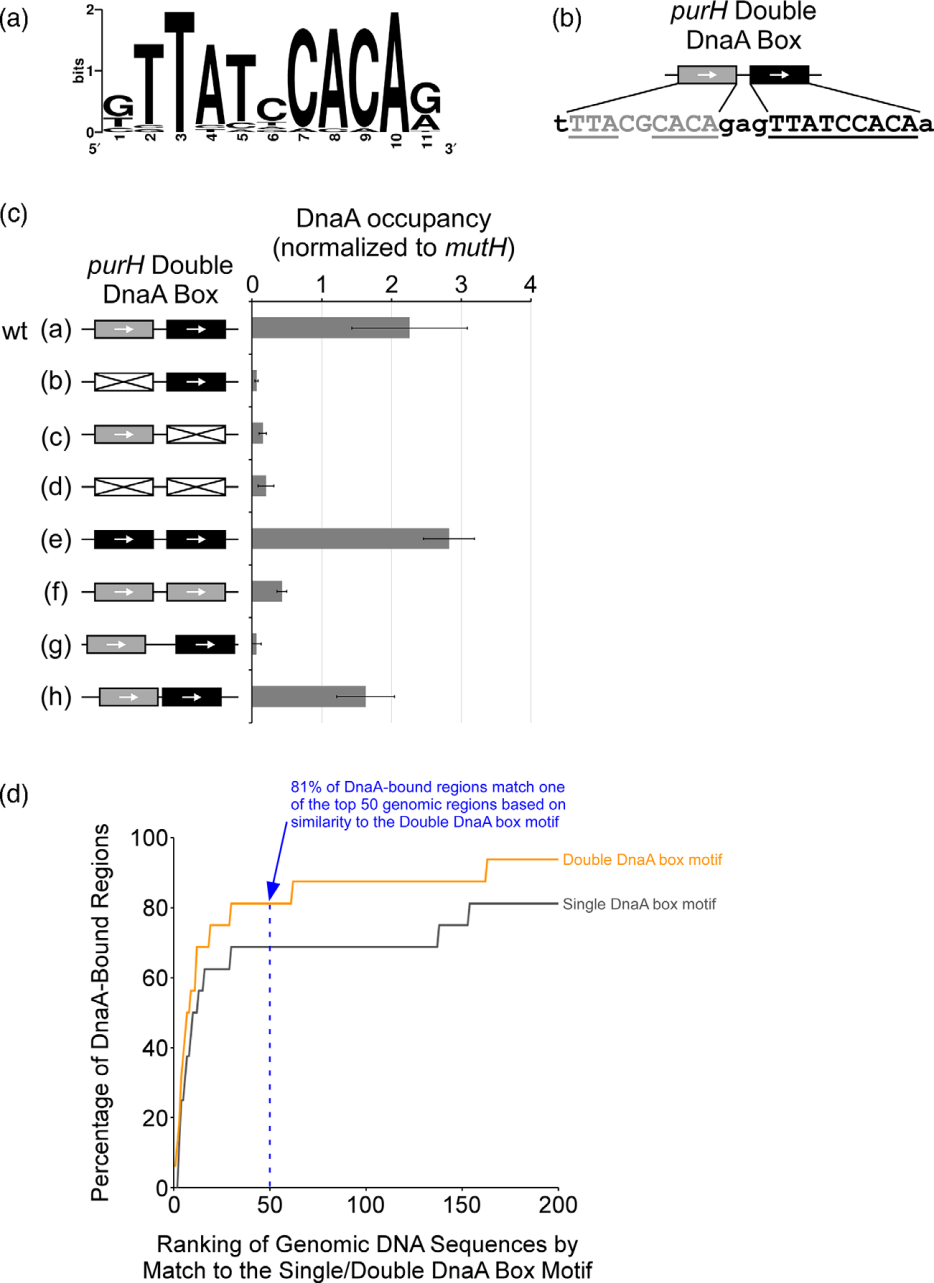
DnaA preferentially binds pairs of DNA sites separated by 2 or 3 bp. (**a**) Sequence logo illustrating the enriched DNA sequence motif associated with DnaA-bound regions. (**b**) Schematic of the double DnaA box upstream of *purH*, with the sequence of each individual DnaA box shown in uppercase. Underlined bases indicate matches to the DnaA box consensus. (**c**) ChIP-qPCR measurements of DnaA association with the *purH* upstream region containing a wild-type or mutated double DnaA box. The nature of the changes is indicated by the schematic to the left of the graph. Black boxes indicate consensus DnaA boxes. Grey boxes indicate DnaA boxes with two base changes from the consensus. Boxes with pairs of diagonal lines indicate fully mutated DnaA boxes. Mutants (**g**, **h**) have altered spacing between the DnaA boxes. Full sequences for the wild-type and mutated boxes are shown in Fig. S2. Note that mutant (**c**) has an additional mutation (C to A substitution) 28 bp downstream of the double DnaA box. (**d**) Genomic DNA sequences were scored by how well they match a single DnaA box motif or a double DnaA box motif (both 2 bp and 3 bp spacing were considered). In the cumulative frequency plot, the *x*-axis indicates the rank of a genomic region with respect to how well it matches the single or double DnaA box motif. The *y*-axis indicates the number of DnaA-bound regions associated with a genomic region of a given rank or better. For example, the top 50 ranked genomic regions, according to their match to a double DnaA box motif, are associated with 81 % of all DnaA-bound regions.

We identified a highly enriched DNA sequence motif in the regions surrounding the stringently selected ChIP-seq peaks (Fig. 1a). This motif is an excellent match to the consensus DnaA box [3]. Strikingly, ChIP-seq peaks upstream of *mutH* (*DARS2*), *polA* and *purH* were associated with pairs of DnaA boxes separated by 3 bp (Table 1), an arrangement we refer to as a ‘double DnaA box’, in contrast to the classical DnaA box, which we refer to as a ‘single DnaA box’. We hypothesized that DnaA binds preferentially to double DnaA boxes rather than single DnaA boxes. Several observations support this hypothesis. First, DnaA can oligomerize [36,37]. Second, DnaA binds cooperatively to high- and low-affinity DNA sites at *oriC* [36,38]; cooperativity may be promoted by the interaction of DnaA with its partner protein DiaA [39]. Third, sequence outside the classical 9 bp DnaA box has been shown to affect DnaA affinity for DNA [3]. Fourth, DnaA binds cooperatively to double DnaA boxes upstream of *nrdA* and *mioC* and at the RK2 plasmid origin [17,40,42]; DnaA binding to each of the double DnaA boxes upstream of *nrdA* and *mioC* is disrupted by mutations in either single site [17,40], and cooperative binding of DnaA to the *mioC* double DnaA box is disrupted by insertion of four additional base pairs between the two single boxes [42]. Fifth, there are 32 instances of a consensus single DnaA box (i.e. TTATCCACA) in the *E. coli* genome, only 13 of which are associated with a DnaA-bound region that we detected using ChIP-seq.

The ChIP-seq peak upstream of *purH* is associated with a putative double DnaA box (Table 1). The upstream DnaA box in the pair has 7/9 matches to the TTATCCACA consensus; the downstream DnaA box is 3 bp from the upstream box and has a perfect match to the consensus (Fig. 1b). To determine the sequence and spacing requirements for DnaA binding to the *purH* double DnaA box, we introduced a series of mutations in the site at its native chromosomal locus. We then used Chromatin Immunoprecipitation coupled with quantitative PCR (ChIP-qPCR) to measure association of DnaA with the site in wild-type and mutant strains. Mutants of the upstream or downstream box, or both, that deviate from the TTATCCACA consensus showed greatly reduced DnaA association (Fig. 1c). By contrast, improving the upstream box such that it is a perfect match to the consensus had no significant effect on DnaA association (Fig. 1c). Insertion of an additional base pair between the two boxes resulted in a large decrease in DnaA association, whereas deleting a base pair between the two boxes had no significant effect on DnaA association (Fig. 1c). Strikingly, these spacing requirements mirror those for DnaA binding to pairs of low-affinity sites at *oriC* [38], suggesting a similar mode of binding.

To test whether the double DnaA box is a better predictor of DnaA binding than the single DnaA box, we searched the *E. coli* genome for matches to either a double or a single DnaA box motif. Given that DnaA binds well to a double DnaA box motif with 2 bp or 3 bp spacing, we allowed for either configuration. We observed close matches to the double DnaA box motif at 15 of the 16 DnaA-bound regions identified by ChIP-seq (Table 1). Overall, the double DnaA box motif was substantially more predictive for DnaA association, as measured by ChIP-seq, than a motif with a single DnaA box (Fig. 1d). The requirement for a double DnaA box likely explains why we did not observe DnaA binding upstream of some previously reported sites that were identified based on matches to the single DnaA box consensus sequence. Of note, the reported site within *guaB* [24,29] is a perfect match to the single DnaA box consensus sequence (i.e. TTATCCACA), but the site was not identified in our motif search using the double DnaA box that identified the 2427 best-scoring sequences. We conclude that DnaA binds to DNA sites *in vivo* predominantly as a dimer, with a preference for sites spaced 2 or 3 bp apart.

## Methods

### Strains and plasmids

Strains AMD029 and CDS100 are unmarked derivatives of MG1655 with C-terminally SPA-tagged copies of *dnaA* and *acpS*, respectively. These strains were constructed by P1 transduction of *kanR*-linked SPA-tagged derivatives of DY330 [43], followed by the removal of the *kan* marker using *flp* recombinase expressed from plasmid pCP20, as previously described [44]. Strain DMF67 (MG1655 *purH::ΔthyA dnaA*-SPA::*kan*) was constructed by P1 transduction of the SPA-tagged *dnaA* from a *kanR*-linked SPA-tagged derivative of DY330 [43] into AMD052 (MG1655 Δ*thyA*) [45]. DMF67 was then used to generate strains DMF69, DMF71, DMF73, DMF75, DMF77, DMF79 and DMF81 that each has mutations in the DnaA-binding site upstream of *purH*, using the Flexible Recombineering Using Integration of *thyA* (FRUIT) method of recombineering, with the native *thyA* gene reintroduced as the last step in the process [45]. In each case, mutations were introduced by recombineering with PCR products generated by Splicing by overlap extension (SOEing) PCR [46]. Note that the strain DMF71 has an additional mutation (C to A substitution) 28 bp downstream of the double DnaA box, likely due to an error in an oligonucleotide used for FRUIT. The *kan* marker associated with the SPA-tagged *dnaA* allele was removed using *flp* recombinase expressed from pCP20, as previously described [44].

### ChIP-seq and peak identification

AMD029 and CDS100 cells were grown in Lysogeny Broth (LB) at 37 °C to an OD_600_ of 0.65. ChIP-seq was performed as has been described previously, using M2 anti-FLAG antibody (Sigma) [47]. Sequences were aligned to the MG1655 genome (NC_000913.2) using Rockhopper, version 2.0.3 [48]. ChIP-seq peaks were identified from replicate DnaA-SPA data, as previously described [49]. ChIP-seq peaks were organized into 90 putative ‘DnaA-bound regions’, defined as loci with one or more ChIP-seq peaks <100 bp from each other. Normalized sequence read coverage was determined for the 201 bp region centred on the central position of each putative DnaA-bound region, for each replicate DnaA-SPA dataset and for each replicate AcpS-SPA dataset. Coverage values for the two DnaA-SPA datasets were averaged, and coverage values for the two AcpS-SPA datasets were averaged. We calculated ‘coverage relative to control’ scores as the ratio of the DnaA-SPA average to the AcpS-SPA average. Putative DnaA-bound regions with a coverage relative to control below 3 were discarded. The putative DnaA-bound region centred at genome position 223 507 was discarded because it is found in repetitive sequence and likely arose from sequence reads originating from the region upstream of *rrsE* (adjacent to the DnaA-bound region upstream of *purH*) being misaligned to the identical sequence upstream of *rrsH*.

### ChIP-qPCR

ChIP-qPCR was performed as described previously [50]. AMD029, DMF069, DMF071, DMF073, DMF075, DMF077, DMF079 or DMF081 cells were grown in LB at 37 °C to an OD_600_ of 0.5–1.0. Two microlitres of M2 anti-FLAG antibody (Sigma) were used for immunoprecipitation. For qPCR, ChIP and input samples were analysed using an ABI 7500 Fast Real-Time PCR machine, as described previously [51]. Enrichment of ChIP samples was calculated for the DnaA-bound region upstream of *purH* (PCR primers: CGCAATAGAAAAATTGCAGA and CGTGGGCAAAATACAGAAAT) and relative to the DnaA-bound region upstream of *mutH* (PCR primers: GCATTGGTTGATCTTTCG and CGGAATTACTACGGGAAAA).

### Quantitative reverse transcription coupled with PCR (q-RTPCR)

Strains MG1655 and AMD029 were grown in LB at 37 °C to an OD_600_ of 0.7–0.8. RNA was purified, DNase I treated and reverse transcribed, as previously described [47]. cDNA was quantified using an ABI 7500 Fast Real-Time PCR machine. Relative changes in cDNA abundance within *nrdA* (PCR primers: CAGCTCCTGCGTACTGAT and CGCTGGGAAACGTATTTA), *polA* (PCR primers: CGGAAGAGGTGGTGAATA and CGCCAGGAATGTTATCAG) and *uvrB* (PCR primers: GTTCGGGGATTGAAAACT and ATTTGTGGAATGGTGACG) were determined by normalizing to the *minD* gene (PCR primers: AACACGCGATAAAGATGC and GGCTTCGTCTGCAAAATA).

### Motif identification and site prediction

Sequences of 101 bp around the centre of each of the 16 DnaA-bound regions were analysed using MEME (version 5.5.1, ‘any number of repetitions’ selected) [52]. The enriched DNA sequences identified as part of the motif using MEME were used to generate a position weight matrix (PWM) that describes the single DnaA box. The best matches to the PWM in the *E. coli* MG1655 genome were identified using FIMO (version 5.5.1) [53]. Matches to the PWM were reported if they had an associated *p*-value ≤0.0001. To search for matches to a double DnaA box with 2 bp spacing, the process was repeated using a PWM where the sequences identified by MEME were combined in tandem. To search for matches to a double DnaA box with 3 bp spacing, the process was repeated using a PWM where the sequences identified by MEME (Fig. S3) were combined in tandem with a random base between the two instances of each sequence.

To evaluate the predictive power of the single DnaA box, we ranked the FIMO results based on the score, with the best matches to the PWMs listed first. We then determined the lowest-ranking FIMO hit that was within 100 bp of the centre of each DnaA-bound region; for the DnaA-bound region upstream of *dnaN*, there were no FIMO hits within 100 bp. To evaluate the predictive power of the double DnaA box motifs, we combined the FIMO results and ranked based on score, with the best matches to the PWMs listed first, and repeated the analysis described earlier.

## supplementary material

10.1099/mic.0.001474 Uncited Fig. S1.

## Abbreviations

ChIP-qPCR: chromatin immunoprecipitation coupled with quantitative PCR
ChIP-seq: chromatin immunoprecipitation coupled with DNA sequencing
*oriC*: origin of replication
PWM: position weight matrix
q-RTPCR: quantitative reverse transcription coupled with PCR
RNR: ribonucleotide reductase
SOEing: splicing by overlap extension
SPA: sequential peptide affinity

## References

[1] Skarstad K, Boye E (1994). The initiator protein DnaA: evolution, properties and function. Biochim Biophys Acta.

[2] Leonard AC, Grimwade JE (2011). Regulation of DnaA assembly and activity: taking directions from the genome. Annu Rev Microbiol.

[3] Schaper S, Messer W (1995). Interaction of the initiator protein DnaA of *Escherichia coli* with its DNA target. J Biol Chem.

[4] Miller DT, Grimwade JE, Betteridge T, Rozgaja T, Torgue J-C (2009). Bacterial origin recognition complexes direct assembly of higher-order DnaA oligomeric structures. Proc Natl Acad Sci U S A.

[5] Felczak MM, Kaguni JM (2004). The box VII motif of Escherichia coli DnaA protein is required for DnaA oligomerization at the *E. coli* replication origin. J Biol Chem.

[6] Kato J, Katayama T (2001). Hda, a novel DnaA-related protein, regulates the replication cycle in *Escherichia coli*. EMBO J.

[7] Nievera C, Torgue J-C, Grimwade JE, Leonard AC (2006). SeqA blocking of DnaA-oriC interactions ensures staged assembly of the *E. coli* pre-RC. Mol Cell.

[8] Campbell JL, Kleckner N (1990). *E. coli* oriC and the dnaA gene promoter are sequestered from dam methyltransferase following the passage of the chromosomal replication fork. Cell.

[9] Kitagawa R, Mitsuki H, Okazaki T, Ogawa T (1996). A novel DnaA protein-binding site at 94.7 min on the *Escherichia coli* chromosome. Mol Microbiol.

[10] Kitagawa R, Ozaki T, Moriya S, Ogawa T (1998). Negative control of replication initiation by a novel chromosomal locus exhibiting exceptional affinity for *Escherichia coli* DnaA protein. Genes Dev.

[11] Kasho K, Katayama T (2013). DnaA binding locus*datA*promotes DnaA-ATP hydrolysis to enable cell cycle-coordinated replication initiation. Proc Natl Acad Sci USA.

[12] Fujimitsu K, Senriuchi T, Katayama T (2009). Specific genomic sequences of *E. coli* promote replicational initiation by directly reactivating ADP-DnaA. Genes Dev.

[13] Saxena R, Fingland N, Patil D, Sharma AK, Crooke E (2013). Crosstalk between DnaA protein, the initiator of *Escherichia coli* chromosomal replication, and acidic phospholipids present in bacterial membranes. Int J Mol Sci.

[14] Messer W, Weigel C (1997). DnaA initiator--also a transcription factor. Mol Microbiol.

[15] Menikpurage IP, Woo K, Mera PE (2021). Transcriptional activity of the bacterial replication initiator DnaA. Front Microbiol.

[16] Quiñones A, Wandt G, Kleinstäuber S, Messer W (1997). DnaA protein stimulates polA gene expression in *Escherichia coli*. Mol Microbiol.

[17] Augustin LB, Jacobson BA, Fuchs JA (1994). *Escherichia coli* Fis and DnaA proteins bind specifically to the nrd promoter region and affect expression of an nrd-lac fusion. J Bacteriol.

[18] Mizushima T, Tomura A, Shinpuku T, Miki T, Sekimizu K (1994). Loss of flagellation in dnaA mutants of *Escherichia coli*. J Bacteriol.

[19] Sass TH, Ferrazzoli AE, Lovett ST (2022). DnaA and SspA regulation of the iraD gene of *Escherichia coli*: an alternative DNA damage response independent of LexA/RecA. Genetics.

[20] Wurihan W, Gezi B, Brambilla E, Wang S, Sun H (2018). DnaA and LexA proteins regulate transcription of the *uvrB* gene in *Escherichia coli*: the role of DnaA in the control of the SOS regulon. Front Microbiol.

[21] Løbner-Olesen A, Atlung T, Rasmussen KV (1987). Stability and replication control of *Escherichia coli* minichromosomes. J Bacteriol.

[22] van den Berg EA, Geerse RH, Memelink J, Bovenberg RA, Magnée FA (1985). Analysis of regulatory sequences upstream of the *E. coli* uvrB gene; involvement of the DnaA protein. Nucleic Acids Res.

[23] Ozaki T, Kumaki Y, Kitagawa R, Ogawa T (2001). Anomalous DnaA protein binding to the regulatory region of the *Escherichia coli* aldA gene. *Microbiology (Reading*).

[24] Tesfa-Selase F, Drabble WT (1992). Regulation of the gua operon of *Escherichia coli* by the DnaA protein. Mol Gen Genet.

[25] Zhou Z, Syvanen M (1990). Identification and sequence of the drpA gene from *Escherichia coli*. J Bacteriol.

[26] Wang QP, Kaguni JM (1989). dnaA protein regulates transcriptions of the rpoH gene of *Escherichia coli*. J Biol Chem.

[27] Gielow A, Kücherer C, Kölling R, Messer W (1988). Transcription in the region of the replication origin, oriC, of *Escherichia coli*: termination of asnC transcripts. *Mol Gen Genet*.

[28] Schaefer C, Messer W (1988). Termination of the *Escherichia coli* asnC transcript. The DnaA protein/dnaA box complex blocks transcribing RNA polymerase. Gene.

[29] Tesfa-Selase F, Drabble WT (1996). Specific binding of DnaA protein to a DnaA box in the guaB gene of *Escherichia coli* K12. Eur J Biochem.

[30] Zeghouf M, Li J, Butland G, Borkowska A, Canadien V (2004). Sequential Peptide Affinity (SPA) system for the identification of mammalian and bacterial protein complexes. J Proteome Res.

[31] Takiff HE, Baker T, Copeland T, Chen SM, Court DL (1992). Locating essential *Escherichia coli* genes by using mini-Tn10 transposons: the pdxJ operon. J Bacteriol.

[32] Lambalot RH, Walsh CT (1995). Cloning, overproduction, and characterization of the *Escherichia coli* holo-acyl carrier protein synthase. J Biol Chem.

[33] Torrents E, Grinberg I, Gorovitz-Harris B, Lundström H, Borovok I (2007). NrdR controls differential expression of the *Escherichia coli* ribonucleotide reductase genes. J Bacteriol.

[34] Mackie GA (2013). RNase E: at the interface of bacterial RNA processing and decay. Nat Rev Microbiol.

[35] Yajnik V, Godson GN (1993). Selective decay of *Escherichia coli* dnaG messenger RNA is initiated by RNase E. J Biol Chem.

[36] Erzberger JP, Mott ML, Berger JM (2006). Structural basis for ATP-dependent DnaA assembly and replication-origin remodeling. Nat Struct Mol Biol.

[37] Weigel C, Schmidt A, Seitz H, Tüngler D, Welzeck M (1999). The N-terminus promotes oligomerization of the *Escherichia coli* initiator protein DnaA. Mol Microbiol.

[38] Rozgaja TA, Grimwade JE, Iqbal M, Czerwonka C, Vora M (2011). Two oppositely oriented arrays of low-affinity recognition sites in oriC guide progressive binding of DnaA during *Escherichia coli* pre-RC assembly. Mol Microbiol.

[39] Keyamura K, Fujikawa N, Ishida T, Ozaki S, Su’etsugu M (2007). The interaction of DiaA and DnaA regulates the replication cycle in *E. coli* by directly promoting ATP DnaA-specific initiation complexes. Genes Dev.

[40] Olliver A, Saggioro C, Herrick J, Sclavi B (2010). DnaA-ATP acts as a molecular switch to control levels of ribonucleotide reductase expression in *Escherichia coli*. Mol Microbiol.

[41] Doran KS, Helinski DR, Konieczny I (1999). A critical DnaA box directs the cooperative binding of the *Escherichia coli* DnaA protein to the plasmid RK2 replication origin. J Biol Chem.

[42] Hansen FG, Christensen BB, Atlung T (2007). Sequence characteristics required for cooperative binding and efficient in vivo titration of the replication initiator protein DnaA in *E. coli*. *J Mol Biol*.

[43] Butland G, Peregrín-Alvarez JM, Li J, Yang W, Yang X (2005). Interaction network containing conserved and essential protein complexes in *Escherichia coli*. Nature.

[44] Datsenko KA, Wanner BL (2000). One-step inactivation of chromosomal genes in *Escherichia coli* K-12 using PCR products. Proc Natl Acad Sci USA.

[45] Stringer AM, Singh N, Yermakova A, Petrone BL, Amarasinghe JJ (2012). FRUIT, a scar-free system for targeted chromosomal mutagenesis, epitope tagging, and promoter replacement in *Escherichia coli* and *Salmonella enterica*. PLoS One.

[46] Horton RM, Cai ZL, Ho SN, Pease LR (1990). Gene splicing by overlap extension: tailor-made genes using the polymerase chain reaction. *Biotechniques*.

[47] Stringer AM, Currenti S, Bonocora RP, Baranowski C, Petrone BL (2014). Genome-scale analyses of *Escherichia coli* and *Salmonella enterica* AraC reveal noncanonical targets and an expanded core regulon. J Bacteriol.

[48] McClure R, Balasubramanian D, Sun Y, Bobrovskyy M, Sumby P (2013). Computational analysis of bacterial RNA-Seq data. Nucleic Acids Res.

[49] Fitzgerald DM, Bonocora RP, Wade JT (2014). Comprehensive mapping of the *Escherichia coli* flagellar regulatory network. *PLoS Genet*.

[50] Bonocora RP, Fitzgerald DM, Stringer AM, Wade JT (2013). Non-canonical protein-DNA interactions identified by ChIP are not artifacts. BMC Genomics.

[51] Wade JT, Reppas NB, Church GM, Struhl K (2005). Genomic analysis of LexA binding reveals the permissive nature of the *Escherichia coli* genome and identifies unconventional target sites. Genes Dev.

[52] Bailey TL, Elkan C (1994). Fitting a mixture model by expectation maximization to discover motifs in biopolymers. Proc Int Conf Intell Syst Mol Biol.

[53] Grant CE, Bailey TL, Noble WS (2011). FIMO: scanning for occurrences of a given motif. *Bioinformatics*.

