# Supplementary material for: Mapping the Escherichia coli DnaA-binding landscape reveals a preference for binding pairs of closely spaced DNA sites
Source: Microbiology (Reading). 2024 Jul 16;170(7):001474. doi: 10.1099/mic.0.001474 (PMC11317965; doi:10.1099/mic.0.001474)
Supplement: Uncited Fig. S1. [file mic-170-01474-s001.pdf]

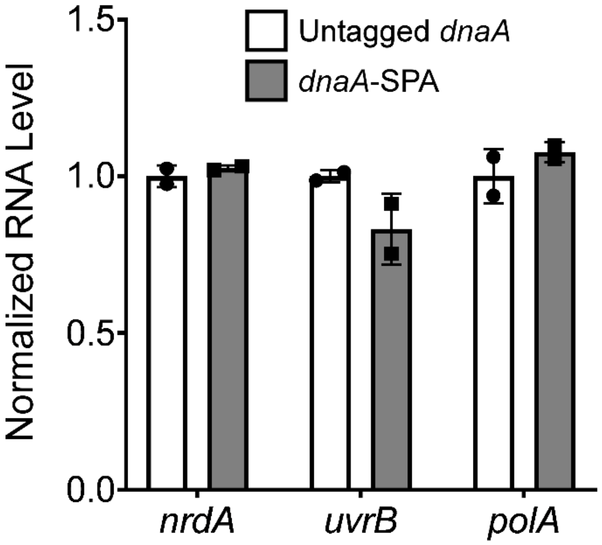

Figure S1. Similar RNA levels of DnaA-regulated genes in untagged and *dnaA*-SPA tagged strains. RNA levels of *nrdA*, *uvrB*, and *polA*, measured by qRT-PCR in an untagged (MG1655) or *dnaA*-SPA strain (AMD029). Values are normalized to those in the untagged strain. Error bars represent ±1 standard deviation from the mean (n = 2).

```
(a)   CCTTTTTACGCACAGA-GTTATCCACAATCAT
(b)   CCTTTaataggtgtGA-GTTATCCACAATCAT
(c)   CCTTTTTACGCACAGA-GaataggtgtATCAT
(d)   CCTTTaataggtgtGA-GaataggtgtATCAT
(e)   CCTTTTTAtcCACAGA-GTTATCCACAATCAT
(f)   CCTTTTTACGCACAGA-GTTAcgCACAATCAT
(g)   CCTTTTTACGCACAGAaGTTATCCACAATCAT
(h)   CCTTTTTACGCACAGG--TTATCCACAATCAT
```

Figure S2. Sequences of wild-type and mutant double DnaA boxes upstream of *purH*. The labeling matches that from Figure 1C. Changes to the wild-type sequence are shown in red. Sequences labeled “b”, “c”, “d”, “e”, “f”, “g”, and “h” correspond to strains DMF69, DMF71, DMF73, DMF75, DMF77, DMF79, and DMF81, respectively. Note that strain DMF71 has an additional mutation (C to A substitution) 28 bp downstream of the Double DnaA box.

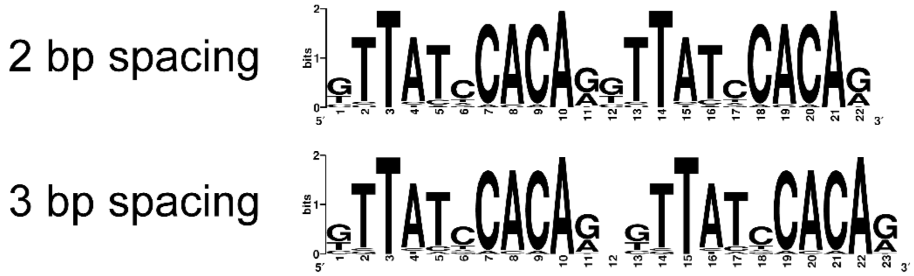

Figure S3. Motifs used to derive position weight matrices used to search for genome sequences that match a Double DnaA box with either 2 bp or 3 bp spacing.
